# THE ASSOCIATION BETWEEN PREOPERATIVE CHEMOTHERAPY AND THE PREVALENCE OF HEPATIC STEATOSIS IN HEPATECTOMY FOR METASTATIC COLORECTAL CANCER

**DOI:** 10.1590/S0102-67202014000200008

**Published:** 2014

**Authors:** Antonio Nocchi KALIL, Gabriela Perdomo CORAL, Félix Antônio Insaurriaga dos SANTOS, Maria Cristina GONZALEZ, Cristiane Becker NEUTZLING

**Affiliations:** Hepatobiliary Cancer Surgery Unit of Santa Rita Hospital and Post-Graduation Program in Hepatology, Hospital Complex of Irmandade Santa Casa of Porto Alegre (Hospital Santa Rita, Complexo Hospitalar da Irmandade Santa Casa de Porto Alegre), Porto Alegre, RS, Brazil.

**Keywords:** Hepatectomy, Chemotherapy, Drug-induced liver injury, Colorectal cancer

## Abstract

**Background:**

Some studies have suggested that preoperative chemotherapy for hepatic colorectal metastases may cause hepatic injury and increase perioperative morbidity and mortality.

**Aim:**

To evaluate the prevalence of hepatic steatosis in patients undergoing preoperative chemotherapy for metastatic colorectal cancer.

**Methods:**

Observational retrospective cohort study in which 166 patients underwent 185 hepatectomies for metastatic colorectal cancer with or without associated preoperative chemotherapy from 2004 to 2011. The data were obtained from a review of the medical records and an analysis of the anatomopathological report on the non-tumor portion of the surgical specimen. The study sample was divided into two groups: those who were exposed and those who were unexposed to chemotherapy.

**Results:**

From the hepatectomies, 136 cases (73.5%) underwent preoperative chemotherapy, with most (62.5%) using a regimen of 5-fluorouracil + leucovorin. A 40% greater risk of cell damage was detected in 62% of the exposed group. The predominant histological pattern of the cell damage was steatosis, which was detected in 51% of the exposed cases. Exposure to chemotherapy increased the risk of steatosis by 2.2 fold. However, when the risk factors were controlled, only the presence of risk of hepatopathy was associated with steatosis, with a relative risk of 4 (2.7-5.9).

**Conclusion:**

Patients exposed to chemotherapy have 2.2 times the risk of developing hepatic steatosis, and its occurrence is associated with the presence of predisposing factors such as diabetes mellitus and hepatopathy.

## Abstract (pt)

**Racional:**

Alguns estudos sugerem que a quimioterapia pré-operatória para metástases hepáticas do câncer colorretal pode causar dano celular e aumentar morbidade e mortalidade.

**Objetivo:**

Analisar a prevalência de esteatose hepática em fígados de pacientes expostos à quimioterapia pré-operatória por metástase de câncer colorretal.

**Métodos:**

O delineamento do estudo foi observacional de coorte retrospectivo, no qual 166 pacientes foram submetidos a 185 hepactectomias por metástase de câncer colorretal, com e sem quimioterapia pré-operatória, no período de 2004 a 2011. Os dados foram extraídos da revisão dos prontuários e da análise do laudo anatomopatológico da parte não tumoral da peça cirúrgica. A amostra foi dividida em grupo exposto e não-exposto à quimioterapia. Os dados foram analisados por programa estatístico Stata 11.2, e aplicado o teste exato de Fischer para análise bivariada, e a regressão de Poisson, para análise multivariada; valores p< 0,05 foram considerados como significativos.

**Resultados:**

Das hepatectomias, 136 casos (73,5%) receberam quimioterapia pré-operatória, e o regime mais utilizado (62,5%) foi 5-fluorouracila+leucovorin. No grupo exposto, a lesão hepatocelular esteve presente em 62% dos casos e correspondeu a risco de 40% em relação ao grupo não-exposto. O padrão histológico da lesão hepatocelular predominante foi a esteatose, em 51% de casos do grupo exposto. A exposição à quimioterapia aumentou em 2,2 vezes a possibilidade de esteatose. Entretanto, quando foram controlados os fatores de risco, somente a hepatopatia prévia esteve associada à presença de esteatose após quimioterapia com risco relativo de 4 (2,7-5,9).

**Conclusões:**

Pacientes expostos à quimioterapia têm risco 2,2 maior de desenvolver esteatose, e sua prevalência está associada à presença de fatores predisponentes, como risco de hepatopatia prévia.

## INTRODUCTION

Liver resections for metastatic colorectal cancer are the only modality of treatment with the potential for long-term survival and the possibility of a cure ^25^. Until the mid-1990s, 5-fluorouracil was the only drug available for treating hepatic metastases from colorectal cancer. With a survival rate below 20% and with no survival advantages, this drug was prescribed as an adjuvant therapy due relapse rates of 60-70% of the cases ^17,18^.

Two potent cytotoxic drugs later emerged: irinotecan and oxaliplatin. The former increased the response rate to 39% and the latter increased the response rate to 51% compared to treatment with 5-fluorouracil alone. The disease-free survival rate improved by approximately 7-8% in three years, and an improvement was noted in the overall survival with different cytotoxicity profiles^11,20^.

Additional benefits arose from the introduction of targeted molecular therapy using monoclonal antibodies such as bevacizumab, cetuximab and panitumumab. Since then, response rates of 66-85%, resection rates of 75%, a 34% 5-year survival rate and a mean survival time of 42-47 months have been achieved, and these results are similar to those obtained with primary resections^21,25^.

Studies have indicated associations between the use of 5-fluorouracil and steatosis, irinotecan and steatohepatitis, and oxaliplatin and sinusoidal dilatation. The data suggest that 5-fluorouracil induces steatosis but not its progression to steatohepatitis, unlike the other two drugs that affect the progression but not the induction^1,22^.

Steatosis is the most common phenotype of liver parenchymal response to cell injury. The damaging effects of steatosis on hepatic resections are most clearly observed in the primary dysfunction of transplanted livers. A 1% increase in fatty infiltration is assumed to correspond to a 1% decrease in the hepatic functional mass ^12,13,24^.

Fatty livers are more susceptible to reperfusion injuries after vascular exclusions such as the Pringle maneuver, which is used to control bleeding in larger hepatic resections^8,24^.

A liver presenting steatosis is softer and more friable, which hinders its parenchymal hemostasis and favors transoperative bleeding. When steatosis is present, the resections must be more limited than initially planned, and the risk of compromised margins and local recurrence increases. Reports have suggested a delayed liver regeneration, a relative risk of postoperative complications between 1.24 and 3.84, a longer stay in the intensive care unit and a 2.78 higher relative risk of mortality^3,8,13,14,24^.

However, the association between chemotherapy and hepatotoxicity is not well supported and exhibits several potential confounding factors. Several conflicting results regarding this association have been reported in the literature as a result of the heterogeneity of the studies^9,15,26^.

Due to these controversies, researchers are studying the prevalence of cell damage in the non-tumor portion of the liver segments resected in the treatment of metastatic colorectal cancer in patients who have or have not been subjected chemotherapy to assess the strength of the association and this is the objective of this paper.

## METHODS

The present study was approved by the Ethics Committee of Irmandade Santa Casa of Porto Alegre under protocol n. 3498/11 from March 11, 2011.

The study utilized an observational retrospective cohort design to analyze a group of consecutive patients who underwent hepatectomy for metastatic colorectal cancer with or without preoperative chemotherapy. The assessment period was from March 2004 to March 2011, in the Hepatobiliary Cancer Surgery Unit, at Santa Rita Hospital of the Hospital Complex of Irmandade Santa Casa of Porto Alegre (Hospital Santa Rita, Complexo Hospitalar da Irmandade Santa Casa de Porto Alegre), Porto Alegre, RS, Brazil.

The strategy consisted of online research using the following search terms: "hepatectomy" ("hepatectomia"), "liver resection" ("ressecção hepática"), "hepatic lobectomy" ("lobectomia hepática"), "hepatic nodulectomy" "(nodulectomia hepática"), "hepatic segmentectomy", ("segmentectomia hepática"), "hepatic bisegmentectomy" ("bissegmentectomia hepática") and "hepatic trisegmentectomy" ("trissegmentectomia hepática") using the resources in the hospital's information technology department.

Using the patient list, the corresponding medical records were reviewed according to the inclusion and exclusion criteria to establish the study sample.

The inclusion criteria were as follows: patients with a) a hepatectomy for metastatic colorectal cancer; and b) exposure, or no exposure, to chemotherapy during the 12 previous months.

The patients were excluded for the following reasons: a) presented with non-colonic hepatic metastasis; b) were subjected to alternative chemotherapy regimens; or c) presented with unresectable colonic or rectal metastasis.

The data from the selected medical records were obtained by a single researcher and were recorded in a printed spreadsheet that was later transcribed into an Excel spreadsheet (Microsoft Office Home and Student 2010, Microsoft Corporation, Redmond, WA 98052 USA). Each hepatectomy was recorded as a new case, so a patient could have more than one entry in the spreadsheet. After the database was finalized, the data were organized, classified, filtered and checked. If conflicts or inconsistency were discovered, the corresponding medical records were manually reviewed.

The as-obtained data were transformed into dichotomous, ordinal and nominal categorical variables, and the two latter were transformed into dichotomous variables for the analysis of association. The data were then analyzed using STATA 11.2 statistical software (Copyright 1985-2009 Stata Corp LP, Texas 77845 USA) . The predictive and outcome variables were organized into a hierarchical structure of the theoretical model as shown in Figure 1.

**FIGURE 1:** Hierarchical structure of the variables for the theoretical model of the multivariate analysis considering p≤0.2

Level 1—Characteristics of the patients
Age; male gender; obesity; comorbidities
Level 2—Characteristics of the chemotherapy
Exposure; regimen
Level 3—Hepatocellular lesion (Outcome)
Liver damage; pathology

The analysis of the association was performed using a Fisher's exact test, and the multivariate analysis was performed with a Poisson regression test: only the variables exhibiting p<0.2 were considered in the hierarchical model shown in Figure 1. Student's t-test was applied to compare the average ages. The results with p<0.05 were deemed significant.

The patient-related predictive variables were as follows: age, gender, obesity, presence of comorbidities.

Among the comorbidities, the variable Diabetes included the two types of diabetes mellitus. The variable Cardiopathy referred to the different identified types of cardiopathy, such as hypertensive, ischemic and congestive cardiopathy. The variable Pneumopathy referred to the cases involving regular smokers, clinical or radiological changes or the use of medication. The variables Nephropathy and Vasculopathy included any reference to the associated diseases. The variable Malnutrition was obtained from the nutritional assessment administered upon admission to the hospital for hepatic resection. The variable Hepatopathy included any reference in the medical records suggestive ou risk of hepatopathy, such as regular alcohol consumption, hepatotoxic drugs (except chemotherapy) and hepatitis B or C^5^.

The chemotherapy- and hepatotoxicity-related variables included exposure, regimen utilized, liver damage and pathology.

The data from the variable Pathology were obtained from the original medical reports that provided a histological description of the non-tumor region from the anatomopathological exam of the surgical specimen. The findings were classified according to four nominal variables: steatosis, vascular, both or normal. The severity of the cell damage could not be quantified. The variable Steatosis included evidence of macro- and microvesicular steatosis and mixed steatosis. The Vascular variable included the presence of sinusoidal ectasia, congestion and regenerative hyperplasia foci as described in the reports. The variable Both corresponded to a concurrent presence of the characteristics from both of the above categories, and the variable Normal was applied when the report described no cell damage or referred to the examination as normal or no changes.

The following variables were considered risk factors for adverse events and are listed with their respective cut-off points: an age equal to or above 60 years, male gender, the presence of obesity and the presence of comorbidities.

## RESULTS

The electronic search identified 178 patients who underwent hepatectomy for metastatic colorectal cancer. Twelve medical records were excluded due to incomplete data, resulting in 166 patients who underwent 185 hepatic resections. From resections assessed, three cases were missing the obesity (1.6%).

Table 1 shows the characteristics of the patients at the time of hepatectomy. The mean age of the patients was 57.6±11.1 years, with 57.3% being male. Approximately 20% of the sample presented with obesity, and the average body mass index (BMI) of the individuals was 26 kg/m^2^. In three cases, the weight or height could not be determined from the medical records. No cases presented dyslipidemia, metabolic syndrome or antioxidant consumption (such as alpha-tocopherol).

**TABLE 1:** Characteristics of patients included at the moment of the hepatectomy (n=185)

Variable			n	%
**Age**				
	< 60		102	55.1
	Mean: 49.6 ± 7.2			
	> 60		83	44.9
	Mean: 67.4 ± 5.8			
**Gender**				
	Female		79	42.7
	Male		106	57.3
**Obesity^a^**				
	B.M.I. < 30		141	77.5
	Mean: 24.6 ± 3			
	B.M.I. ≥ 30		41	22.5
	Mean: 33.2 ± 4.6			
**Comorbidities**				
	No		119	64.3
	Yes		66	35.7
**Types of comorbidities^b^**				
	Cardiopathy		85	45.9
	Hepatopathy		39	21.1
	Diabetes		27	14.6
	Pneumopathy		24	12.9
	Malnutrition		8	4.3
	Nephropathy		5	2.7
	Vasculopathy		2	1.1
**Exposure to chemotherapy**				
	No		49	26.49
	Yes		136	73.51
**Chemotherapy regimen**				
	5FU/LV		85	62.5
	+Oxaliplatin		39	28.7
	+Irinotecan		5	3.7
	+Target therapy		7	5.1
**Liver damage**				
	No		78	42.2
	Yes		107	57.8
**Pathology**				
	Steatosis		41	22.2
	Vascular		46	24.9
	Both		20	10.8
	No hepatoxicity		78	42.1

Obesity=BMI ≥30 kg/m2; BMI=body mass index; 5FU/LV=5-fluorouracil + leucovorin;

a 3 unreported cases;

b more than one event/case

Comorbidities were present in 35.7% of the cases with cardiopathy as the most prevalent being present in 45.9% of the cases. Risk of Hepatopathy was present in 21.1% of the cases, and the only case of cirrhosis was excluded from the sample due to other incomplete data (this case was among the 12 rejected medical records).

Preoperative chemotherapy was applied to 73.5% of the cases, and the regimen used most often (in 62.5% of the cases) was based on 5-fluorouracil + leucovorin, followed by a 5-fluorouracil + leucovorin + oxaliplatin regimen in 28.7% of the cases.

The records assessment did not allow for any differentiation between the neoadjuvant chemotherapy and conversion or rescue chemotherapy. In addition, the number of cycles and the rest period between the administration of chemotherapy and the surgical resection could not be identified.

Liver damage was observed in 57.8% of the cases. Among patients with liver damage, 38.3% had only steatosis, and 43.9% displayed exclusive vascular lesions. Among the cases with cell damage, 61 (57%) exhibited steatosis.

Nineteen cases of rehepatectomies were observed normal parenchyma at the first resection was observed in six cases, steatosis was detected in two cases and vascular lesions were detected in four cases during the second resection. Among the 61 cases with steatosis, 8.2% exhibited recurrence and underwent a rehepatectomy. Among the 124 cases that did not exhibit steatosis, 11.3% exhibited recurrence (p=0.6). The mean BMI was 26.5 in the patients who underwent rehepatectomy and 26.8 (p=0.8) in the patients without recurrence.

The analysis of Table 2 provides the association between the studied variables and the use of chemotherapy as a risk factor. The use of preoperative chemotherapy in cases of rectal tumors and advanced stages was more frequent in patients below 60 years of age. The average age of the exposed group was four years below that of the unexposed group.

**TABLE 2:** Profiles of the patients exposed to or not exposed to preoperative chemotherapy at the time of hepatectomy for metastatic colorectal cancer (n=185)

Level	Variable		No CT n = 49 (%)	CT n = 136 (%)	RR (CI 95%)		p value*	
1	Age							
		≥ 60 years	28 (57)	55 (40)	0.7 (0.5–0.9)		**0.04**	
		Mean (years)	60.2 ± 10.4	56.6 ± 11.2			0.02**	
	Gender							
		Male	29 (59)	77 (56)	0.9 (0.7–1.3)		0.8	
	Obesity^a^							
		Yes	10 (21)	31 (23)	1.1 (0.6–2)		1	
	Comorbidities							
		Yes	30 (61)	89 (65)		1 (0.8–1.4)	0,6	
3	Liver damage							
		Yes	22 (45)	85 (62)	1.4 (1–1.9)			**0.04**
	Pathology							
		Steatosis^b^	8 (23)	53 (51)	2.2 (1.2–4.2)			**0.005**
		Vascular^c^	17 (38)	49 (49)	1.2 (0.8–1.9)			**0.2**

CT=chemotherapy; RR=relative risk; CI=confidence interval;

* Fisher's exact test;

** Student's t-test; Obesity=BMI >30 kg/m2;

a 3 unreported cases;

b n=139 (41 steatosis + 20 both + 78 normal);

c n=144 (46 vascular + 20 both + 78 normal)

Cell damage and the presence of steatosis were more prevalent in the exposed group. The relative risks were 1.4 (1.0-1.9) and 2.2 (1.2-4.2), respectively. No association was noted between the use of preoperative chemotherapy and the other variables of levels 1.

Table 3 shows the analysis of the association in the presence of steatosis. Patients under 60 years of age exhibited a higher incidence. Diabetes mellitus, hepatopathy and exposure to chemotherapy were risk factors for steatosis.

**TABLE 3:** Analysis of the association with steatosis (n=61)

Level	Variable		Steatosis n = 61 (%)		RR (CI 95%)	p value*
1	Age					
		< 60 years	41 (40)		1	
		≥ 60 years	20 (24)		0.6 (0.4–0.9)	**0.02**
	Gender					
		Female	25 (31)		1	
		Male	36 (34)		1.1 (0.7–1.6)	0.7
	Obesity^a^					
		< 30	44 (31)		1	
		≥ 30	17 (41)		1.3 (0.8–2)	0.2
	Comorbidities					
		No		12 (18)	1	
		Yes		49 (41)	2.3 (1.3–3.9)	**0.001**
	Diabetes					
		No	46 (29)		1	
		Yes	15 (55)		1.9 (1.3–2.9)	**0.01**
	Hepatopathy					
		No	28 (19)			
		Yes	33 (84)		4.4 (3.1–6.3)	**< 0.001**
2	Chemotherapy					
		No	9 (18)		1	
		Yes	52 (38)		2.1 (1.1–3.9)	**0.01**

RR=unadjusted relative risk; CI=confidence interval;

* Fisher's exact test; Obesity=BMI ≥ 30kg/m^2^;

a 3 unreported cases

Table 4 shows the regression analysis and indicates that the presence of hepatopathy was the only risk factor for steatosis when the two other variables were controlled.

**TABLE 4:** Multivariate analysis of the risk factors for steatosis (n=185)

Variable	RR (CI 95%)	p value*
Diabetes mellitus	1.3 (0.8–2)	0.2
Hepatopathy	4 (2.7–5.9)	**< 0.001**
Exposure to chemotherapy	1.6 (0.9–2.7)	0.1

* Poisson regression test

## DISCUSSION

Advances in preoperative chemotherapy have significantly improved the results of hepatic resections^2,18^.

This strategy is based on several factors, including decrease in lesion volume, preserving a greater part of the non-tumor liver, reducing the size of the resections and the level of compromised margins, control of the micrometastases at distances not detected via the imaging methods, and improvement in the progression-free survival^2,4,9,20^.

However, this approach is followed by the hepatotoxicity associated with chemotherapy, which leads to a clinical paradox: this therapy transforms an unresectable patient into a resectable one, but it can lead to a level of cell damage that can preclude resection? Achieving a balance between the aggressive resection strategies and the aggressive chemotherapy strategies is a challenge for the multidisciplinary teams^1^.

Chemotherapy-associated hepatotoxicity presents two different histological patterns: non-alcoholic fatty liver disease with its steatosis and steatohepatitis spectrum and vascular lesions^24^.

In the present study, cell damage was noted in 62% of the group exposed to chemotherapy versus 44% of the unexposed group which is in agreement with the data by Pessaux et al.^23^.

The average occurrence of hepatic steatosis in the population (overall population) is 20% and ranges from 6.3 to 33% depending on the assessment method and region studied. Hepatic steatosis is the most common response of the liver to cellular injury and is estimated to be present in more than 20% of candidates for hepatic resection^6,24^. The present study demonstrated an occurrence of steatosis of 51% of the group exposed to preoperative chemotherapy and 23% in the unexposed group, a result similar to that of the review study by Pilgrim et al. ^24^ who found a that the exposed group had a risk of steatosis at least two times higher than the unexposed group.

The steatosis-independent risk factors are obesity, diabetes mellitus type 2, dyslipidemia, age and male gender^6^. The present study identified exposure to chemotherapy, diabetes mellitus and the presence of previous history exposure cell damage as the risk factors for steatosis in the bivariate analysis The patients of 60 years of age or more exhibited a lower occurrence of steatosis. The increased exposure to chemotherapy in the group under 60 years of age could explain this difference. Pawlik et al.^22^, Spelt et al.^27^ and Cook et al.^10^ also reported a wider use of chemotherapy in younger patients. The increased exposure to chemotherapy in the group under 60 years of age could explain this difference. Pawlik et al.^22^, Spelt et al. ^27^ and Cook et al.^10^ also reported a wider use of chemotherapy in younger patients. Wolf et al.^29^ reported rates of 12% for diabetes mellitus and 48% for cardiopathy; these results are similar to those in the present study, which found percentages of 14.6% and 45.9%, respectively. The prevalence of risk of hepatopathy in the present study was 21.1%, which is similar to that obtained by Brouquet et al.^5^, who reported a level of 28.7% in 2009. The present study found no identified cases of dyslipidemia.

In the present study, 73.5% of the cases were exposed to preoperative chemotherapy, a trend similar to the current management of hepatic metastases from colorectal cancer. In 2012, Viganò et al.^28^ grouped their sample of 376 patients into three sub-groups according to date for analysis: from 1985 to 1994, from 1995 to 2000 and from 2001 to 2005. Preoperative chemotherapy was not offered to the first group, but was prescribed for 19.1% of the second group and 43.8% of the last group. The case study by Wolf et al.^29^ found that 65% of patients underwent this treatment, and Pawlik et al.^22^ found levels of 72.2%, which are similar to those of the present study.

An association between the use of 5-fluorouracil + leucovorin with steatosis and oxaliplatin and the occurrence of vascular lesions has been reported by some authors^1,9^. The most common regimens used in the present study were as follows: 5-fluorouracil + leucovorin in 62.5% of patients and oxaliplatin in 28.7% of the cases. Similarly, Chan et al.^7^ demonstrated that 5-fluorouracil + leucovorin was used in 54% of the cases prior to 2003 and 5-fluorouracil + leucovorin + oxaliplatin was used in 23.8% of the cases after 2003. In the case study by Spelt et al.^27^, oxaliplatin was employed in 73.7% of the cases, following the current trend for the first-line of treatment for advanced cases.

The design of the present study did not distinguish between the group exposed to the preoperative chemotherapy and the group exposed to the adjuvant regimens with primary colorectal tumor resection. These tumors, when in stages II and III, have indications for regimens based on 5-fluorouracil + leucovorin for six months^19^, which explains the predominance of this protocol and the steatosis as a histological pattern of injury.

In the present study, 19 cases involved rehepatectomy. Among this subgroup, 1/3 presented normal histology at the first resection and cell damage at the second. Two cases presented an onset of steatosis, and four cases vascular lesions, possibly due to the addition of oxaliplatin^19^.

In the present case series, the presence of steatosis did not increase the risk of local recurrence in contrast to the findings of Hamady et al.^14^, who reported that steatosis is an independent risk factor for local liver recurrence following resections of hepatic metastases from colorectal cancer.

In the present study, the prevalence of cell damage was 40% higher in the exposed group with steatosis as the predominant histological pattern with a level of 51%, corresponding to a 2.2 higher risk over that of the unexposed group.

These findings are in agreement with reports by Kooby et al.^16^ from a study of 485 patients under a similar chemotherapy regimen that demonstrated a relative risk of 1.7% (p<0.01). Pawlik et al.^22^ published a study in 2007 with a research design similar to the present study. The authors found an odds ratio of 5 (CI 95%=1.5-23.8) for steatosis in the exposed group and concluded that preoperative chemotherapy could be a predictive factor independent of steatosis.

However, Robinson et al.^26^ published a metanalysis and systematic review in 2012 and found no association between the presence of steatosis and the use of preoperative chemotherapy. The authors, nonetheless, observed a marked heterogeneity among the studies (from I^2^ =19% to I^2^ =74%) and discrepancies in the assessments by the pathologists.

The design selected for the present study did not allow for differentiation between the cases of steatosis and steatohepatitis. Considering that irinotecan was used only in 3.7% of the cases, one can assume that this study limitation did not have an effect on the results or conclusions.

The second manifestation of chemotherapy-associated hepatotoxicity are the vascular lesions, which in the present study were also more prevalent in the exposed group, although no significant association could be established with the use of oxaliplatin.

Robinson et al.^26^ demonstrated a risk of 4.36% (CI 95%=1.36-13.97); p< 0.01, I^2^=77%. The authors, however, observed a high heterogeneity due to differences in the protocol.

Wolf et al.^29^ reported that cell damage would only be significantly higher in the exposed group when the other factors, such as a BMI≥25 kg/m^2^ or diabetes mellitus, were present. Their results were similar to the present study, whose strength of association between the chemotherapy exposure and steatosis disappeared when the diabetes mellitus variables, and especially the hepatopathy variables, were controlled.

This observation corroborates the hypothesis that cell damage occurs through a mechanism that involves two consecutive molecular insults, the first of which is the presence of one risk factor such as obesity, diabetes mellitus or previous history of hepatopathy. This condition would lead to an excess of fatty acids in the hepatocyte and an increase in the oxygen reactive species. The oxidative stress would hinder the action of the mitochondria and would render the liver cells susceptible to a second stage of injury. This second stage would involve the exposure to the cytotoxic chemotherapy drugs with an additional release of free radicals^9^.

Thus, one can conclude, through the causal network, that the action of preoperative chemotherapy could initiate cell damage, but it alone is not a sufficient cause^24,29^.

The sufficient cause would any prior histological changes in the parenchyma due to the first stage of the insult^24,29^.

The present study demonstrated some limitations that are inherent to the retrospective design. One of these is the lack of control over the nature and quality of the variables. The collection of data from the pathology reports did not follow a research protocol.

In addition, once organized, the data regarding the chemotherapy regimen, dose, number of cycles, time of exposure and rest period could aid in the interpretation of the effect of preoperative chemotherapy on the liver parenchyma but obtaining such data was impossible. Despite the difficulties and limitations in the data collection, the findings of the present study were similar to those that follow the same design and are found in the literature^22^.

## CONCLUSION

The actual effect on the liver of preoperative chemotherapy based on cytotoxic drugs is controversial and inferential, and reports, such as the present study, generally show only a weak association with little consistency. Thus, one can conclude that cell damage from preoperative chemotherapy only occurs when the liver parenchyma was already exposed to the first insult. Recognizing the strength of the effects and the interaction of the causes can allow for a better understanding of the impact of preoperative chemotherapy on the hepatotoxicity patterns and can lead to improved therapeutic strategies.
